# Diagnostic discordance in amyloidosis: lessons from proteomic amyloid typing

**DOI:** 10.1093/ckj/sfag297

**Published:** 2026-09-03

**Authors:** Manuel Lopes da Silva, Telma Pais, João Boavida, Carolina Branco, Pedro Pinheiro, Gonçalo Justino, José António Lopes, Natacha Rodrigues

**Affiliations:** Department of Nephrology and Renal Transplantation, Unidade Local de Saúde Santa Maria, Lisbon, Portugal; Faculdade de Medicina da Universidade de Lisboa, Lisbon, Portugal; Department of Nephrology and Renal Transplantation, Unidade Local de Saúde Santa Maria, Lisbon, Portugal; Faculdade de Medicina da Universidade de Lisboa, Lisbon, Portugal; Faculdade de Medicina da Universidade de Lisboa, Lisbon, Portugal; Department of Pathological Anatomy, Unidade Local de Saúde Santa Maria, Lisbon, Portugal; Department of Nephrology and Renal Transplantation, Unidade Local de Saúde Santa Maria, Lisbon, Portugal; Faculdade de Medicina da Universidade de Lisboa, Lisbon, Portugal; Centro de Química Estrutural – Institute of Molecular Sciences, Instituto Superior Técnico, Universidade de Lisboa, Lisbon, Portugal; Departamento de Engenharia Química, Instituto Superior Técnico, Universidade de Lisboa, Lisbon, Portugal; Escola Superior de Tecnologia do Barreiro, Instituto Politécnico de Setúbal, Lavradio, Portugal; Centro de Química Estrutural – Institute of Molecular Sciences, Instituto Superior Técnico, Universidade de Lisboa, Lisbon, Portugal; Escola Superior de Tecnologia do Barreiro, Instituto Politécnico de Setúbal, Lavradio, Portugal; Department of Nephrology and Renal Transplantation, Unidade Local de Saúde Santa Maria, Lisbon, Portugal; Faculdade de Medicina da Universidade de Lisboa, Lisbon, Portugal; Department of Nephrology and Renal Transplantation, Unidade Local de Saúde Santa Maria, Lisbon, Portugal; Faculdade de Medicina da Universidade de Lisboa, Lisbon, Portugal

To the Editor,

Amyloidosis encompasses several disorders characterized by tissue deposition of misfolded proteins, which lead to multiorgan damage. Diagnosis requires tissue biopsy for accurate amyloid typing, which is essential because treatment strategies differ substantially according to the precursor protein involved. However, the coexistence of monoclonal gammopathies and amyloidosis may create diagnostic uncertainty, particularly when conventional typing techniques yield inconclusive results or when clinical and pathological findings appear discordant [1, 2].

Proteomic amyloid typing by mass spectrometry (MS) is increasingly considered the reference standard for amyloid classification because of its high sensitivity and specificity, especially when compared to conventional techniques such as immunofluorescence (IF) and immunohistochemistry (IHC) [3, 4].

We describe five diagnostically challenging patients in whom MS established or revised amyloid subtype despite misleading or inconclusive conventional studies, directly influencing clinical management (Table 1). For these tissue biopsies, a non-laser microdissection approach was used, followed by proteomic analysis with ultra-high-performance liquid chromatography coupled with high-resolution MS [5].

**Table 1:** Amyloid MS cases

	Patient 1	Patient 2	Patient 3	Patient 4	Patient 5
**Age (years)**	78	86	61	68	78
**Sex**	Female	Female	Male	Male	Male
**Presentation**	Nephrotic syndrome	Nephrotic syndrome	Peripheral neuropathy and cardiac involvement	CKD and subnephrotic proteinuria	Severe AKI requiring hemodialysis
**Serum creatinine (mg/dl)**	1.88	2.48	1.05	1.65	11.5
**uACR (mg/g)**	3597	7400	3.7	1168	Unmeasurable
**uPCR (mg/g)**	4082	9927	40.5	2101	Unmeasurable
**Monoclonal protein**	None detected	IgM λ	None detected	IgG λ	IgA κ
**Bone marrow plasma cells (%)**	2.4	Patient refused	Not performed	11	26.4
**Initial diagnosis**	AA amyloidosis	Monoclonal gammopathy	ATTR amyloidosis	Indolent myeloma	IgA κ multiple myeloma
**Tissue biopsy**	Kidney	Kidney	Salivary gland	Kidney	Abdominal fat
**Congo red**	Positive	Positive	Positive	Positive	Positive
**Conventional typing**	AA on IHC	No light-chain restriction	Negative IF/IHC	Negative IF/IHC	No light-chain restriction
**MS amyloid subtype**	AL λ	AL λ	AL λ	AL λ	AL λ
**Discordance identified**	AA vs AL	MGUS without restriction	ATTR vs AL	Indolent myeloma vs AL	κ clone vs λ amyloid
**Initial management**	No hematologic treatment	Watchful waiting	Planned tafamidis	Watchful waiting	Supportive care
**Post-MS management**	Dara-VCD	BDR	Hematologic reassessment	Dara-VRD	No treatment initiated
**Impact of MS on management**	Change in therapy	Treatment initiation	Diagnostic reassessment	Initiation of clone-directed therapy	Diagnostic reclassification

AKI, acute kidney injury; ATTR, transthyretin amyloidosis; BDR, bortezomib, rituximab, dexamethasone; CKD, chronic kidney disease; Dara-VCD, daratumumab, bortezomib, cyclophosphamide, dexamethasone; Dara-VRD, daratumumab, bortezomib, lenalidomide, dexamethasone; uACR, urinary albumin-creatinine ratio; uPCR, urinary protein-creatinine ratio.

Patient 1: a 78-year-old woman presenting with nephrotic syndrome was initially diagnosed with AA amyloidosis on kidney biopsy. In the absence of an underlying inflammatory disorder, MS was performed and identified AL λ amyloidosis, resulting in treatment modification.

Patient 2: an 86-year-old woman with nephrotic syndrome and an IgM λ monoclonal gammopathy had amyloid deposits without light-chain restriction on conventional tests. MS confirmed AL λ amyloidosis and supported the decision to initiate treatment.

Patient 3: a 61-year-old man with peripheral neuropathy, cardiac involvement, positive family history, and pathogenic testing for transthyretin amyloidosis, underwent salivary gland biopsy demonstrating amyloid deposits. Despite a clinical picture strongly suggestive of ATTR amyloidosis, MS identified AL λ amyloidosis, raising the possibility of concomitant ATTR and AL λ amyloidosis, prompting hematologic reassessment. Considering the significant prevalence of ATTR hereditary amyloidosis in Portugal along with positive genetic testing and clinical presentation, tafamidis was initiated, maintaining a close surveillance of treatment response and plasma cell dyscrasia.

Patient 4: a 68-year-old man with chronic kidney disease, proteinuria, and an IgG λ monoclonal gammopathy was initially managed with a watchful waiting strategy, as bone marrow biopsy showed 11% plasma cells without myeloma-defining events. Kidney biopsy demonstrated Congo red–positive amyloid deposits with negative IF and IHC, even after pronase digestion. After MS, AL λ amyloidosis diagnosis was established, which led to treatment initiation.

Patient 5: a 78-year-old man with IgA κ multiple myeloma and severe acute kidney injury requiring dialysis had amyloid deposits identified on abdominal fat biopsy. MS demonstrated AL λ amyloid, highlighting discordance between the detected plasma cell clone and the amyloid subtype. Several mechanisms may explain this clone–subtype discordance, including amyloid fibril formation by a quantitatively minor but structurally unstable light chain, lower specificity of conventional techniques in detecting truncated immunoglobulins or biclonal expression [6]. Owing to rapid clinical deterioration, treatment was not initiated.

In all cases, MS either established or revised the amyloid subtype when conventional tests were inconclusive or inconsistent with the clinical presentation. Notably, the presence of a monoclonal gammopathy did not reliably predict amyloid subtype in three patients, and one patient with genetically confirmed ATTR amyloidosis was ultimately found to have concomitant AL λ amyloidosis. These findings illustrate the limitations of relying solely on conventional typing techniques or monoclonal protein characterization in patients with suspected amyloidosis [1, 2, 7].

The coexistence of monoclonal gammopathy and non-AL amyloidosis represents a well-recognized diagnostic challenge, particularly in older patients. Conversely, the identification of a monoclonal protein may lead clinicians to incorrectly assume AL amyloidosis in the absence of definitive typing. Our cases demonstrate that clinicopathological discordance should prompt further investigation, as both conventional typing techniques and plasma cell clone characterization may fail to accurately identify the amyloid precursor protein.

As access to proteomic amyloid typing expands, MS may play an increasingly important role in resolving diagnostic uncertainty and assist in guiding appropriate subtype-directed therapy [3, 4].
